# A Systematic Review and Meta-Analysis Evaluating the Role of GLP-1 Receptor Agonists in Substance Use Disorders

**DOI:** 10.7759/cureus.111641

**Published:** 2026-06-28

**Authors:** Indrani Sarma, Krishna P Biswas, Prerna Jagdish, Praveen K, Kashif Akhtar Ahmed, Dibyajyoti Saikia

**Affiliations:** 1 Pharmacology, All India Institute of Medical Sciences, Guwahati, Guwahati, IND; 2 Dentistry, All India Institute of Medical Sciences, Guwahati, Guwahati, IND; 3 Anatomy, Employees' State Insurance Corporation (ESIC) Beltola, Guwahati, IND; 4 Anatomy, All India Institute of Medical Sciences, Guwahati, Guwahati, IND; 5 Orthopedic Surgery, All India Institute of Medical Sciences, Guwahati, Guwahati, IND

**Keywords:** addiction, alcohol use disorder, exenatide, glp-1 receptor agonists, grade, randomized controlled trial, semaglutide, substance use disorder, tobacco use disorder

## Abstract

GLP-1 receptor agonists (GLP-1RAs) have shown preclinical effects on reward-seeking behavior across several substance classes, but human randomized controlled trial (RCT) evidence remains limited. This systematic review and meta-analysis evaluated the effects of GLP-1RAs on substance-use outcomes in adults with substance use disorders. Electronic databases and trial registries were searched from inception to April 30, 2026, for parallel-group or crossover RCTs comparing any GLP-1RA with placebo or control. Outcomes included days without alcohol consumption, cigarettes smoked per day (CPD), and Fagerström Test for Nicotine Dependence (FTND) scores. Five RCTs met the inclusion criteria, including 764 participants with follow-up ranging from six to 52 weeks. Three RCTs contributed alcohol-related outcomes, and four contributed tobacco-related outcomes. Random-effects meta-analysis showed no statistically significant effect on days without alcohol consumption (MD −1.96 days, 95% CI −17.97 to 14.05; I² = 74%), CPD (MD −0.55 cigarettes/day, 95% CI −1.76 to 0.65; I² = 45%), or FTND score (MD 0.02, 95% CI −0.32 to 0.36; I² = 0%). Risk-of-bias assessment using the Cochrane Risk-of-Bias (RoB) 2 tool rated three trials as low risk overall and two as having some concerns. Certainty of evidence assessed using GRADE (Grading of Recommendations, Assessment, Development and Evaluations) ranged from low to moderate. Current pooled RCT evidence does not demonstrate a statistically significant benefit of GLP-1RAs for alcohol or tobacco use outcomes. A possible signal of benefit in patients with comorbid alcohol use disorder and obesity should be interpreted as hypothesis-generating because of the small number of trials, clinical heterogeneity, and imprecision. Adequately powered, long-duration, agent-specific RCTs using standardized substance-use outcomes are required before clinical translation can be recommended.

## Introduction and background

Substance use disorders (SUDs) are major contributors to preventable morbidity and mortality worldwide. Alcohol use disorder (AUD) contributes substantially to global disease burden, while tobacco use remains responsible for millions of deaths annually [1,2]. Although evidence-based pharmacotherapies are available, including naltrexone and acamprosate for AUD, buprenorphine for opioid use disorder, and varenicline and bupropion for tobacco cessation, long-term abstinence and sustained remission remain difficult to achieve for many patients [3,4]. This persistent treatment gap has encouraged investigation of novel neurobiological targets that may influence craving, reinforcement, and relapse.

GLP-1RAs, or glucagon-like peptide-1 receptor agonists, were developed primarily to treat type 2 diabetes mellitus (T2DM) and, more recently, have been approved for obesity. They have emerged as an unexpected class of candidates for SUD treatment [5]. GLP-1 receptors are present not only in peripheral metabolic tissues but also in brain regions involved in reward processing and motivated behavior, including mesolimbic and limbic circuits. Preclinical studies suggest that GLP-1RA administration may reduce alcohol intake, nicotine-related reward, and drug-seeking behaviors in animal models [6,7]. GLP-1 is co-released with dopamine in the nucleus accumbens (NAc) during reward processing, and exogenous GLP-1RA administration attenuates dopaminergic responses to rewarding stimuli [8,9].

Extensive preclinical evidence across rodent and non-human primate models has demonstrated that systemic or intracranial GLP-1RA administration reduces voluntary alcohol self-administration, attenuates nicotine-induced dopamine release, decreases cocaine- and amphetamine-reinstatement, and blunts sucrose preference, effects attributed to direct modulation of mesolimbic circuitry rather than secondary metabolic changes [10,11]. However, these findings provide biological plausibility rather than proof of clinical efficacy, and human randomized evidence remains limited. We therefore conducted this systematic review and meta-analysis to evaluate the pooled effects of GLP-1RAs on alcohol and tobacco use outcomes across available RCTs.

## Review

Methods

Protocol and Registration

This systematic review and meta-analysis were conducted in accordance with the Preferred Reporting Items for Systematic Reviews and Meta-Analyses (PRISMA 2020) guidelines [12,13]. The protocol was prospectively registered on PROSPERO (CRD420261419602). This was an inter-institutional individual collaborative study.

Eligibility Criteria

Studies were considered eligible if they included adults aged 18 years or older with a diagnosis of any substance use disorder defined according to the Diagnostic and Statistical Manual of Mental Disorders, Fifth Edition (DSM-5) or the International Classification of Diseases, 11th Revision (ICD-11) criteria [14,15]. Eligible interventions included any licensed or investigational glucagon-like peptide-1 receptor agonist, including but not limited to exenatide, semaglutide, dulaglutide, liraglutide, tirzepatide, albiglutide, or efpeglenatide, administered at any dose, by any route, and for any duration. Eligible comparators were placebo, either matched or unmatched, or an active pharmacological control. Studies were required to report at least one pre-specified or post-hoc validated measure of substance use, including quantity consumed, frequency of use, abstinence rates, craving scores, or validated dependence severity scales. Alcohol and tobacco use disorders were included because GLP-1 signaling has been hypothesized to influence shared reward, craving, and reinforcement pathways across substances. Only randomized controlled trials, with any level of blinding and published as full-text articles in any language, were included. Exclusion criteria were non-randomized or quasi-randomized designs, preclinical studies, observational, cohort, or case-control data, conference abstracts without accessible full-text data, and studies reporting exclusively metabolic or weight outcomes without any substance use measure.

Search Strategy

A systematic and exhaustive literature search was carried out across seven electronic databases and trial registries, encompassing MEDLINE (accessed via PubMed), EMBASE (accessed via Ovid), the Cochrane Central Register of Controlled Trials (CENTRAL), PsycINFO, ClinicalTrials.gov, the WHO International Clinical Trials Registry Platform (ICTRP), and EudraCT, with searches covering all records from each database's inception date up to and including April 30, 2026. The search strategy was constructed by combining controlled vocabulary terms - specifically Medical Subject Headings (MeSH) for MEDLINE and Emtree for EMBASE - with supplementary free-text keywords organized across three conceptual domains. The first domain captured GLP-1 receptor agonists and included the following agents and terminology: exenatide, semaglutide, dulaglutide, liraglutide, tirzepatide, albiglutide, efpeglenatide, GLP-1, and glucagon-like peptide-1. The second domain targeted substance use disorders and encompassed terms related to alcohol use disorder, alcoholism, heavy drinking, tobacco, nicotine, smoking, cocaine, opioids, cannabis, and stimulants. The third domain incorporated validated randomized controlled trial search filters to restrict retrieval to experimental study designs. Neither language restrictions nor date limitations were imposed at any stage of the search process. To ensure comprehensive retrieval and minimise the risk of missing eligible studies, reference lists of all included trials, pertinent systematic reviews, and previously published meta-analyses were independently hand-searched for additional potentially relevant citations. 

Study Selection and Data Extraction

Two independent reviewers screened the titles and abstracts. Full-text review was conducted for all potentially eligible records. Disagreements were resolved through structured discussion and arbitration by a third reviewer. All data were collected in a standardized form, like study identifiers, study design and setting, participant characteristics, GLP-1RA agent and dose, co-interventions, follow-up duration, outcome definitions, reported means, standard deviations, and sample sizes.

Risk-of-Bias Assessment

The revised Cochrane Risk-of-Bias (RoB) 2.0 tool was used to assess risk of bias and visualized using the robvis package in R [16,17]. It was independently done by two reviewers across five pre-specified domains. Each domain was rated 'Low risk', 'Some concerns', or 'High risk' [18]. An overall study-level RoB judgement was assigned per RoB 2.0 algorithm. Discrepancies were resolved by consensus.

Statistical Analysis

Continuous outcomes (HDD, CPD, FTND) were pooled as mean differences (MD) where common measurement scales were used, or as standardized mean differences (SMD) where scales differed. For dichotomous outcomes (smoking abstinence), risk ratios (RR) were pre-specified.

The degree of statistical heterogeneity across included studies was evaluated using two complementary approaches. First, the Cochran Q test was applied, with a significance threshold set at p < 0.10 to account for the characteristically limited statistical power of this test when the number of contributing studies is small. Second, the I² statistic was calculated to express the proportion of total variability in effect estimates attributable to genuine between-study differences rather than sampling error alone. Interpretation of I² values followed pre-specified benchmarks established a priori: values below 25% were classified as indicative of low heterogeneity, values between 25% and 50% as moderate, values between 50% and 75% as high, and values exceeding 75% as very high heterogeneity [19]. In instances where the I² statistic exceeded 50%, pooled effect estimates were interpreted with heightened caution, acknowledging the presence of likely substantial variance across study populations, interventions, or outcome measurement approaches. The between-study variance parameter (Tau²), representing the true dispersion of underlying effect sizes across trials, was derived using the DerSimonian-Laird estimation method. Because only a small number of studies contributed to each pooled outcome, we considered the potential limitations of DerSimonian-Laird random-effects estimation. Hartung-Knapp-type adjustment may provide more conservative confidence intervals in small meta-analyses; therefore, pooled estimates were interpreted cautiously, with emphasis on confidence intervals, heterogeneity, and certainty of evidence rather than statistical significance alone.

Funnel plots were used to assess publication bias for outcomes with ≥3 studies, with Egger's regression test planned where ≥10 studies were available per outcome (threshold not reached in this analysis). Pre-specified subgroup analyses were planned for: GLP-1RA agent class, comorbid obesity status (BMI ≥30 vs mixed/unselected), and SUD type (AUD vs. tobacco). All analyses were performed in RevMan 5.4 (Cochrane Collaboration, Copenhagen, Denmark) [20].

Certainty of Evidence Assessment

Certainty of evidence for each pre-specified outcome was assessed using the GRADE (Grading of Recommendations, Assessment, Development and Evaluations) framework, following the Cochrane Handbook and GRADEpro GDT (Guideline Development Tool) [21,22]. All RCT evidence begins at HIGH certainty and is downgraded by one level (−1, serious concern) or two levels (−2, very serious concern) for each of five domains: risk of bias, inconsistency (unexplained heterogeneity), indirectness (PICO or Population, Intervention, Comparator, Outcome mismatch), imprecision (wide confidence intervals, optimal information size not met), and publication bias. Certainty may be upgraded for a large effect size (+1 for RR >2.0 or OR >5.0 with no plausible confounding), a dose-response gradient (+1), or where all plausible residual confounding would reduce an apparent effect (+1).

Results

Study Selection

**Figure 1:**
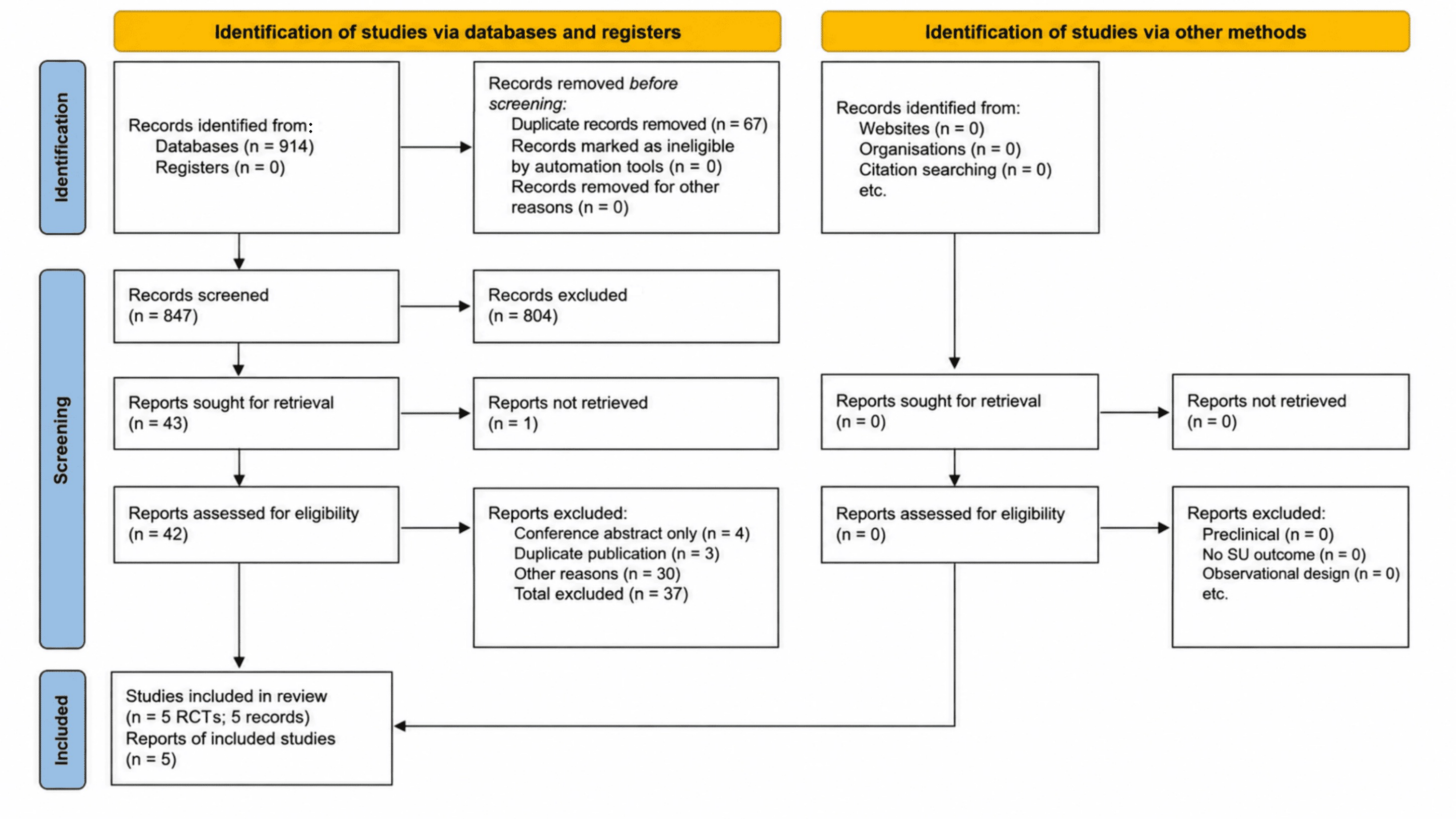
Study selection process following PRISMA guidelines PRISMA: Preferred Reporting Items for Systematic Reviews and Meta-Analyses

The systematic database search identified 847 unique records after de-duplication from 914 records. Screening of titles and abstracts excluded 804 records; 42 full-text articles were retrieved for eligibility assessment. Of these, 37 were excluded. A total of five RCTs (n = 764 participants) were included in the quantitative synthesis. PRISMA flowchart of the study selection process is illustrated in Figure 1.

Characteristics of Included Studies

The five included RCTs were published between 2021 and 2026 and conducted across five countries [23-27]. GLP-1RAs evaluated were: semaglutide, exenatide, and dulaglutide. Three RCTs targeted AUD (Alcohol Use Disorder), and two targeted tobacco use disorder. The mean participant age ranged from 38 to 54 years. Most trials used intention-to-treat (ITT) analysis with multiple imputation for missing data. Table 1 provides full study-level baseline characteristics.

**Table 1:** Baseline characteristics of included studies ALC: alcohol; AUD: alcohol use disorder; AUDIT: Alcohol Use Disorders Identification Test; AUDIT-C: Alcohol Use Disorders Identification Test-Consumption; BMI: body mass index; CBT: cognitive behavioral therapy; Con: control group; CPD: cigarettes per day; DSM-5: Diagnostic and Statistical Manual of Mental Disorders, Fifth Edition; FTND: Fagerström Test for Nicotine Dependence; HDD: heavy drinking days; Int: intervention group; M: male; NRT: nicotine replacement therapy; rand.: randomized; SC: subcutaneous; WHO: World Health Organization

Sl no.	Study (author)	Country	Addiction type	GLP-1 drug & dose	n Int (rand.)	n Con (rand.)	Age Int mean±SD	Age Con mean±SD	Sex % Male Int / Con	BMI Int mean±SD	BMI Con mean±SD	Baseline addiction severity (Int)	Baseline addiction severity (Con)	Duration & background treatment
1	Klausen MK et al. (2022) [23]	Denmark (Copenhagen)	Alcohol use disorder (AUD)	Exenatide 2 mg/week SC (once-weekly)	62	65	44.7±11.5	45.4±10.9	Int: 77.4% M Con: 73.8% M	25.8±4.4	25.9±4.2	HDD 30 days: 63.0±23.5% AUDIT: 22.3±6.2 Alcohol: 857±610 g/30d DSM-5 AUD: severe 79%	HDD 30 days: 62.0±24.5% AUDIT: 22.3±5.7 Alcohol: 858±583 g/30d DSM-5 AUD: severe 79%	26 weeks + standard CBT (both arms)
2	Hendershot CS et al. (2025) [24]	USA (North Carolina academic center)	Alcohol use disorder (AUD) + Tobacco subgroup (27%)	Semaglutide 0.25→0.5→ 1.0 mg/week SC (dose escalation)	24	24	34.8±9.2	34.2±8.9	Int: ~52% M Con: ~48% M	31.6±5.8	31.4±6.1	Drinks/wk: ~28.5±18.3 HDD: ~45% AUDIT-C: ~9.8 ALC craving: moderate Non-treatment seeking	Drinks/wk: ~27.8±17.9 HDD: ~42% AUDIT-C: ~9.5 ALC craving: moderate Non-treatment seeking	9 weeks (dose escalation) No background pharmacotherapy
3	Klausen MK et al. (2026) [25]	Denmark (Copenhagen, single center)	Alcohol use disorder (AUD) + Obesity (BMI ≥30)	Semaglutide 0.25→0.5→1.0→ 1.7→2.4 mg/week SC (dose escalation)	~54	~54	~48.1±10.8	~47.9±11.2	Int: ~70% M Con: ~68% M	~36.2±5.1	~36.4±5.3	HDD baseline: ~70% AUDIT >15 ≥6 HDD/30 days WHO risk level: high/very high (treatment seeking)	HDD baseline: ~70% AUDIT >15 ≥6 HDD/30 days Treatment seeking	26 weeks + standard CBT (both arms)
4	Langsfield et al. (2023) [26]	Switzerland (Basel, single center)	Tobacco use disorder (primary outcome)	Dulaglutide 1.5 mg/week SC (once-weekly)	128	127	53.2±10.5	52.8±10.2	Int: 54.7% M Con: 52.0% M	29.5±5.2	29.8±5.6	CPD: 18.2±9.5 FTND: 4.9±2.1 Smoking ≥10 CPD for ≥1 year Motivated to quit	CPD: 18.8±9.3 FTND: 5.0±2.0 Smoking ≥10 CPD for ≥1 year Motivated to quit	12 weeks active; 52 weeks follow-up + varenicline + behavioral counselling (both)
5	Yammine L et al. (2021) [27]	USA (Houston, Texas)	Tobacco use disorder (Nicotine dependence)	Exenatide 2 mg/week SC (once-weekly)	42	42	44.2±11.0	43.1±11.2	Int: 64% M Con: 67% M	31.5±5.3	31.8±5.9	CPD: 17.8±7.8 FTND: 5.4±2.1 Pre-diabetes and/or BMI ≥25 kg/m² ≥10 CPD for ≥1 yr Treatment seeking	CPD: 18.1±8.5 FTND: 5.3±2.3 Pre-diabetes and/or BMI ≥25 kg/m² ≥10 CPD for ≥1 yr Treatment seeking	6 weeks + NRT 21 mg patch + brief counselling (both arms)

Risk of Bias

**Figure 2:**
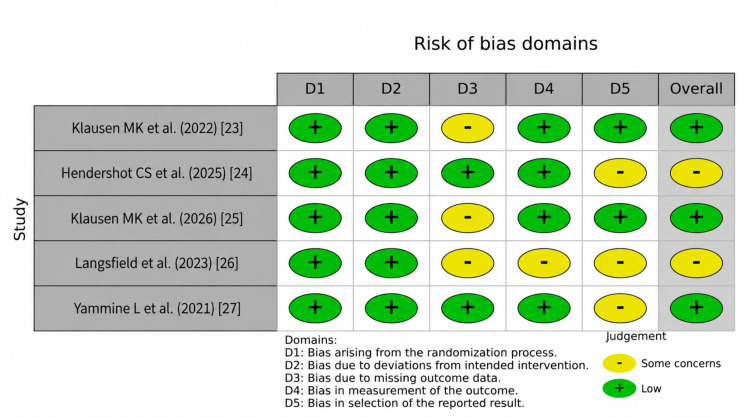
Risk-of-bias assessment of the included studies Risk-of-bias assessment of included randomized controlled trials using the Cochrane Risk-of-Bias 2 (RoB 2) tool, generated using the robvis package in R [16,17]. Each study was assessed across five domains: D1, bias arising from the randomization process; D2, bias due to deviations from intended interventions; D3, bias due to missing outcome data; D4, bias in measurement of the outcome; and D5, bias in selection of the reported result [23-27].

Figure 2 presents the RoB 2.0 domain-level traffic light summary for all five included RCTs [23-27]. All studies achieved low risk ratings for D1 (randomization process), all five were also rated low for D2 (deviations from intended intervention), and D3 (missing outcome data) was the domain of greatest concern across the review. Three studies received 'some concerns'. D4 (outcome measurement) was rated low in all five studies. D5 (selection of reported results) raised 'some concerns' in two studies. No study was rated 'High' risk of bias overall.

Days Without Alcohol Consumption

**Figure 3:**
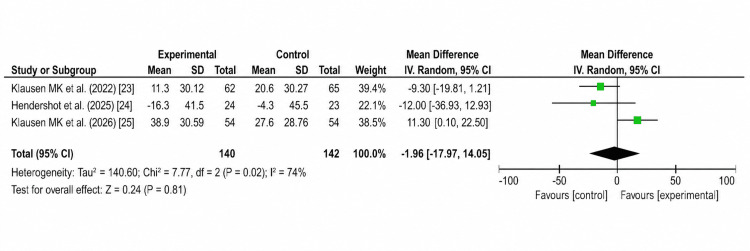
Forest plot showing the effect of GLP-1 receptor agonists versus control on days without alcohol consumption The outcome was analysed as a continuous variable using mean difference (MD) with an inverse-variance random-effects model. Each green square represents the study-specific effect estimate, with the size of the square proportional to study weight; horizontal lines indicate 95% confidence intervals [23-25]. The diamond represents the pooled mean difference. The pooled estimate was MD −1.96 days, 95% CI −17.97 to 14.05. Substantial heterogeneity was observed, with I² = 74%.

For the outcome of days without alcohol consumption, three studies involving 282 participants were included in the meta-analysis, with 140 participants in the GLP-1 receptor agonist group and 142 in the control group [23-25]. The total pooled sample was experimental n=140, control n=142 (N=282) (Figure 3).

The pooled random-effects estimate showed no statistically significant difference between GLP-1RAs and control (MD −1.96 days, 95% CI −17.97 to 14.05; p = 0.81). Heterogeneity was substantial (I² = 74%), indicating that the pooled estimate should be interpreted with caution. The wide confidence interval is compatible with both possible benefit and possible lack of benefit; therefore, the current evidence supports uncertainty rather than a definitive treatment effect.

Exploration of heterogeneity sources suggests three primary drivers, such as GLP-1RA agents differ substantially in half-life, receptor binding kinetics, and likely CNS bioavailability, population obesity status, and outcome measurement [23-25]. Formal subgroup meta-analyses by agent and BMI status were not feasible with three studies.

Cigarettes Per Day (CPD)

**Figure 4:**
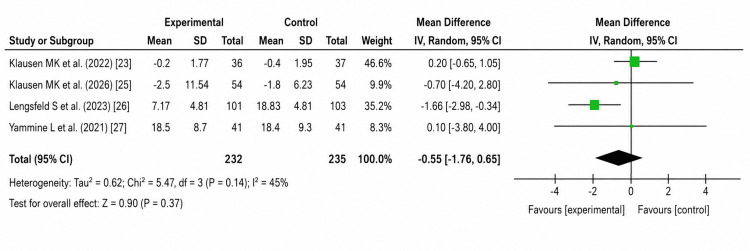
Forest plot for the effect of GLP-1 receptor agonists on cigarettes smoked per day Forest plot showing the pooled effect of GLP-1 receptor agonists compared with control on change in cigarettes smoked per day. The outcome was analysed as a continuous variable using mean difference (MD) with an inverse-variance random-effects model. Four studies comprising 232 participants in the experimental group and 235 participants in the control group were included [23,25-27]. The pooled estimate showed no statistically significant difference between groups (MD −0.55 cigarettes/day, 95% CI −1.76 to 0.65; Z = 0.90, p = 0.37). Moderate heterogeneity was observed across studies (Tau² = 0.62; Chi² = 5.47, df = 3, p = 0.14; I² = 45%).

Four RCTs contributed CPD data. Total pooled sample: experimental n=232, control n=235 (N=467) (Figure 4).

Four RCTs involving 467 participants contributed data for cigarettes smoked per day. The pooled estimate showed no statistically significant reduction with GLP-1RAs compared with control (MD −0.55 cigarettes/day, 95% CI −1.76 to 0.65; p = 0.37). Heterogeneity was moderate (I² = 45%). Although one individual study favoured GLP-1RA treatment, the pooled effect was small, statistically non-significant, and of uncertain clinical importance [26].

Fagerström Test for Nicotine Dependence (FTND)

**Figure 5:**
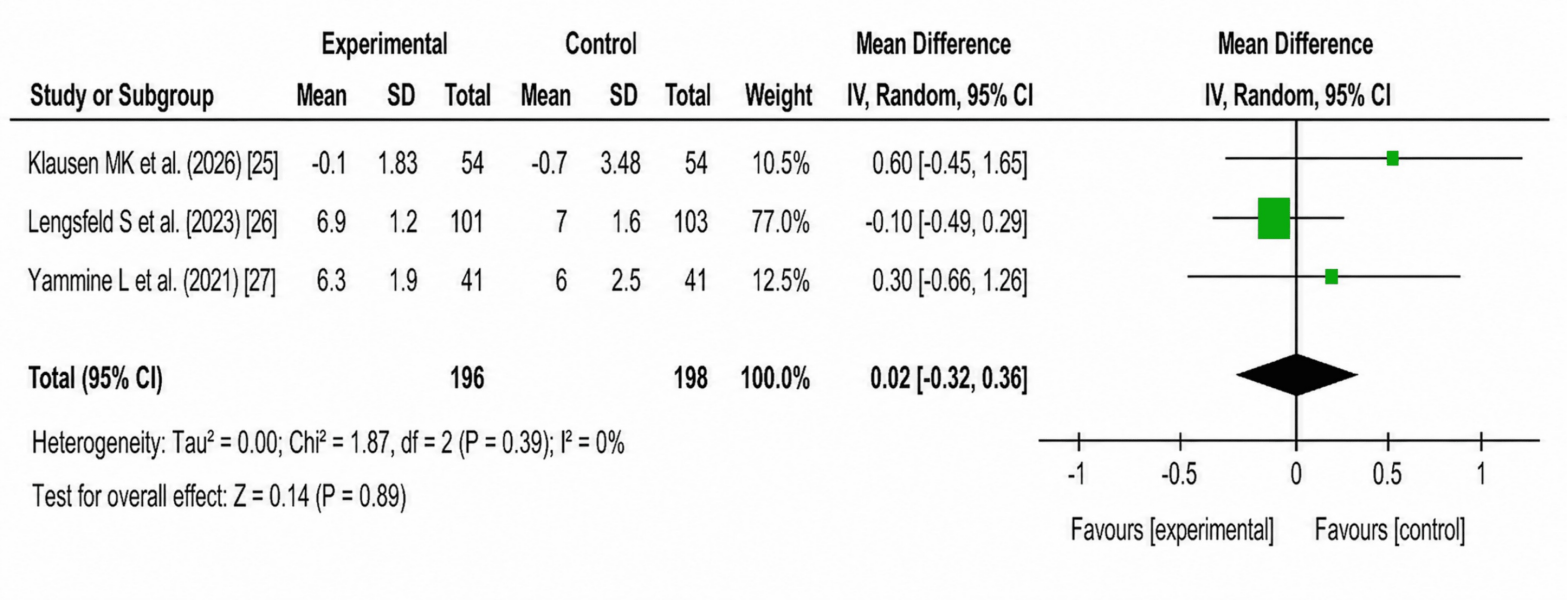
Forest plot of Fagerström Test for Nicotine Dependence (FTND) scores comparing experimental and control groups The plot shows study-specific and pooled mean differences using the inverse-variance random-effects model. Green squares were used to represent individual study estimates; the square size was proportional to study weight. The diamond represents the pooled effect estimate. Across the three studies, Klausen MK et al. (2026), Lengsfeld S et al. (2023), and Yammine L et al. (2021) [25-27], there was no significant difference between experimental and control groups (pooled mean difference 0.02, 95% CI −0.32 to 0.36; p = 0.89), with no observed heterogeneity (I² = 0%).

Three RCTs provided FTND data [25-27] (Figure 5).

Three RCTs involving 394 participants contributed FTND data. The pooled random-effects estimate showed no statistically significant difference between GLP-1RAs and control (MD 0.02 points, 95% CI −0.32 to 0.36; p = 0.89), with no observed heterogeneity (I² = 0%). The magnitude of effect was negligible and does not suggest a clinically meaningful reduction in nicotine dependence severity.

Publication Bias

**Figure 6:**
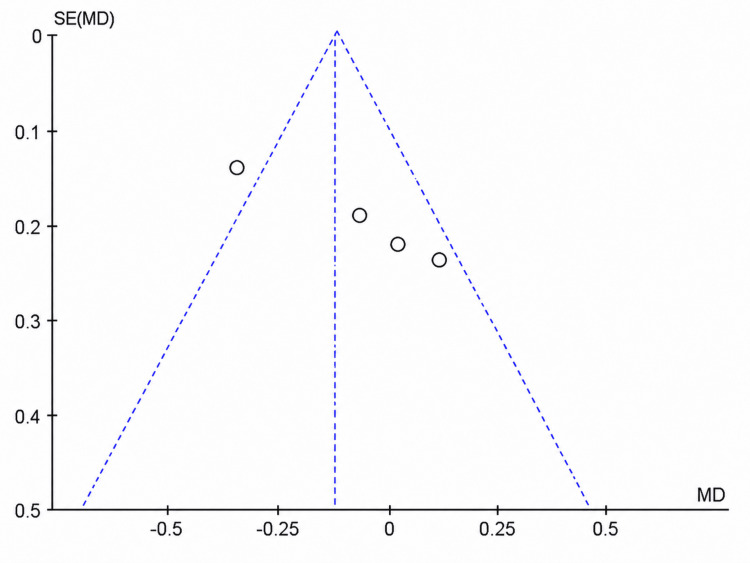
Funnel plot assessing small-study effects Each circle represents an included study [23,25-27]. The vertical blue dashed line represents the pooled effect estimate, while the diagonal dashed lines indicate the pseudo 95% confidence limits around the pooled estimate.

The funnel plot for the study-related outcomes included four studies and showed a mildly asymmetric distribution of effect estimates (Figure 6).

Publication bias and small-study effects could not be reliably assessed because only a small number of studies were included. Although mild visual asymmetry was observed, this finding is not interpretable with confidence when fewer than ten studies are available. Formal asymmetry testing was therefore not performed, and no conclusion regarding publication bias was drawn.

Certainty of Evidence

GRADE was used to find out the certainty of evidence. It was found to be low to moderate as shown in the summary of findings table (Table 2). The summary of findings table included three continuous outcomes: days without alcohol consumption, cigarettes smoked per day, and FTND score. For days without alcohol consumption, three RCTs involving 282 participants contributed data [23-25]. GLP-1RAs showed no clear improvement compared with placebo/control, with a mean difference of 1.96 days lower in the intervention group (95% CI: 17.97 lower to 14.05 higher). The certainty of evidence was rated as moderate.

**Table 2:** GLP-1RAs compared to placebo/control for substance use disorder (GRADE assessment) Patient or population: Substance use disorder Intervention: GLP-1RAs Comparison: Placebo/control GRADE (Grading of Recommendations, Assessment, Development and Evaluations) Working Group grades of evidence: High certainty: The true effect is very likely to be close to the estimated effect. Moderate certainty: The true effect is likely to be close to the estimated effect, but there is a possibility that it is substantially different. Low certainty: Confidence in the estimated effect is limited; the true effect may be substantially different from the estimated effect. Very low certainty: Confidence in the estimated effect is very limited; the true effect is likely to be substantially different from the estimated effect. CI: confidence interval; MD: mean difference; FTND: Fagerström Test for Nicotine Dependence

Outcomes	Anticipated absolute effects (95% CI)		Relative effect (95% CI)	No. of participants (studies)	Certainty of the evidence (GRADE)
	Risk with Placebo/control	Risk with GLP-1RAs			
Days without alcohol consumption (Days without alcohol consumption) follow-up: 6 weeks	The mean days without alcohol consumption was 0 days	MD 1.96 days higher (17.97 higher to 14.05 higher)	-	282 (3 RCTs)	⊕⊕⊕○ Moderate
Cigarettes smoked per day	The mean cigarettes smoked per day was 0	MD 0.55 higher (1.76 higher to 0.65 higher)	-	467 (4 RCTs)	⊕⊕⊕○ Moderate
FTND score	The mean FTND score was 0	MD 0.02 higher (0.32 lower to 0.32 higher)	-	394 (3 RCTs)	⊕⊕⊕○ Moderate

For cigarettes smoked per day, four RCTs including 467 participants were included [23,25-27]. The pooled estimate suggested 0.55 fewer cigarettes smoked per day with GLP-1RAs compared with placebo/control; however, the confidence interval crossed the line of no effect (95% CI: 1.76 lower to 0.65 higher). The certainty of evidence was rated as moderate.

For the FTND score, three RCTs, including 394 participants, contributed data [25-27]. There was no meaningful difference between GLP-1RAs and placebo/control, with a mean difference of 0.02 points higher in the intervention group (95% CI: 0.32 lower to 0.32 higher). The certainty of evidence was rated as moderate. Overall, moderate-certainty evidence suggests that GLP-1RAs do not demonstrate a clear clinically important benefit for alcohol abstinence days, cigarettes smoked per day, or nicotine dependence severity based on the currently available randomized evidence.

Discussion

Principal Findings

This review found that current randomized evidence does not demonstrate a statistically significant pooled effect of GLP-1RAs on alcohol or tobacco use outcomes. For alcohol-related outcomes, interpretation was limited by substantial heterogeneity, wide confidence intervals, and differences in study populations, including the presence or absence of obesity. For tobacco-related outcomes, pooled effects on cigarettes smoked per day and FTND score were small and not clinically persuasive. Overall, the findings support continued investigation of GLP-1RAs in SUDs but do not provide sufficient evidence for clinical use specifically for alcohol or tobacco use disorders [24].

Mechanistic Basis and Pharmacological Considerations

The biological rationale for studying GLP-1RAs in addiction is supported mainly by preclinical evidence [8]. GLP-1 signaling may influence reward processing, craving, and reinforcement through effects on mesolimbic pathways [9,10]. However, the extent to which these mechanisms translate into clinically meaningful reductions in substance use in humans remains uncertain. Therefore, mechanistic explanations should be interpreted as hypothesis-generating and not as proof of clinical efficacy.

Heterogeneity: Implications and Resolution

The I² of 74% in the alcohol free days analysis is the most important interpretative challenge of this meta-analysis. Statistical heterogeneity of this magnitude indicates that the between-study variance substantially exceeds within-study sampling variation, and the true treatment effects across studies are likely genuinely different. In this context, the pooled MD of −1.96 represents an average across incomparable populations and agents rather than a clinically meaningful estimate applicable to any specific patient group.

The alcohol outcome showed substantial heterogeneity, suggesting that differences in GLP-1RA agent, dose, treatment duration, comorbid obesity, baseline severity, background behavioral treatment, and outcome definition may have influenced study-specific effects. With only three alcohol-related trials, formal subgroup analyses were not reliable. Future research should prioritize adequately powered, agent-specific RCTs with standardized outcomes. Individual participant data meta-analysis may also help clarify whether factors such as BMI, baseline AUD severity, or metabolic status modify treatment response.

Tobacco Use Disorder

The tobacco findings present in the largest and most precise estimate contributed a null result for smoking abstinence, with a confidence interval tightly straddling the line of no effect. This pattern suggests GLP-1RAs engage metabolic but not addiction-relevant reward circuits sufficiently to alter smoking behavior [9,10]. In contrast, a point estimate indicating a large effect (RR 1.70) produced notable heterogeneity and limited the interpretability of any pooled effect.

Limitations

This meta-analysis has some limitations. First, the small number of included RCTs (n = 5 in quantitative synthesis) substantially limits statistical power for subgroup analyses, increases the influence of each study on pooled estimates, and precludes assessment of small-study effects via formal regression tests. Second, marked clinical and methodological heterogeneity limits the clinical interpretability of pooled estimates, particularly for alcohol outcomes. Third, most included trials were short (6-26 weeks), so whether GLP-1RA effects on substance use are maintained with longer treatment or after discontinuation is entirely unknown. Assessment of publication bias was also limited because the number of included studies was too small for reliable funnel plot interpretation or formal asymmetry testing.

Clinical and research implications

At present, GLP-1RAs should not be recommended specifically for the treatment of alcohol or tobacco use disorders outside research settings. In patients who already meet approved metabolic indications for GLP-1RA therapy, any possible substance-use benefit should be regarded as preliminary and hypothesis-generating [25-27]. Future trials should be adequately powered, longer in duration, and designed around standardized substance-use outcomes, including validated craving measures, abstinence outcomes, biomarkers where relevant, and prespecified subgroup analyses by obesity or metabolic status.

Recent evidence syntheses and large observational studies have expanded interest in GLP-1RAs for substance use disorders. A recent systematic review including both preclinical and clinical studies reported consistent preclinical effects across several substance classes, but concluded that clinical evidence remains preliminary, heterogeneous, and limited by small sample sizes and short follow-up. Large observational data have also suggested associations between GLP-1RA use and lower risk of substance-use-related outcomes, but such findings remain non-randomized and cannot establish clinical efficacy. Therefore, these studies support the biological and clinical rationale for further trials but do not change the central interpretation of the present meta-analysis that current randomized evidence remains insufficient for clinical recommendations [28-30].

## Conclusions

This systematic review and meta-analysis of five RCTs found no statistically significant pooled effect of GLP-1RAs on alcohol or tobacco use outcomes. The evidence remains limited by the small number of trials, heterogeneity across populations and interventions, short follow-up, and imprecision. A possible benefit in selected patients with comorbid AUD and obesity remains hypothesis-generating and requires confirmation in adequately powered trials. Current evidence is insufficient to recommend GLP-1RAs specifically for SUD treatment, although further agent-specific RCTs with standardized outcomes are warranted.
